# Supplementary material for: LECT2 drives haematopoietic stem cell expansion and mobilization via regulating the macrophages and osteolineage cells
Source: Nat Commun. 2016 Sep 6;7:12719. doi: 10.1038/ncomms12719 (PMC5025878; doi:10.1038/ncomms12719)
Supplement: Supplementary Information — Supplementary Figures 1-8 [file ncomms12719-s1.pdf]

Supplementary Information:

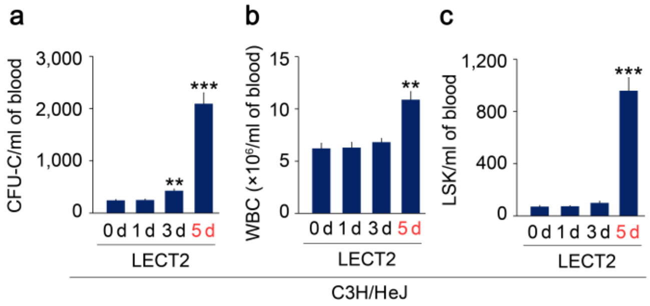

Supplementary Figure 1 LECT2 increases the mobilization of LSK cells in C3H/HeJ mice. (**a**) The number of CFU-Cs per millilitre of mouse blood. (**b**) The number of WBCs in the blood. (**c**) The number of LSK cells in BM. *n* = 5. The data represent means±s.e.m. The data are representative of two independent experiments. ***P*< 0.01, ****P*< 0.001 using one-way ANOVA.

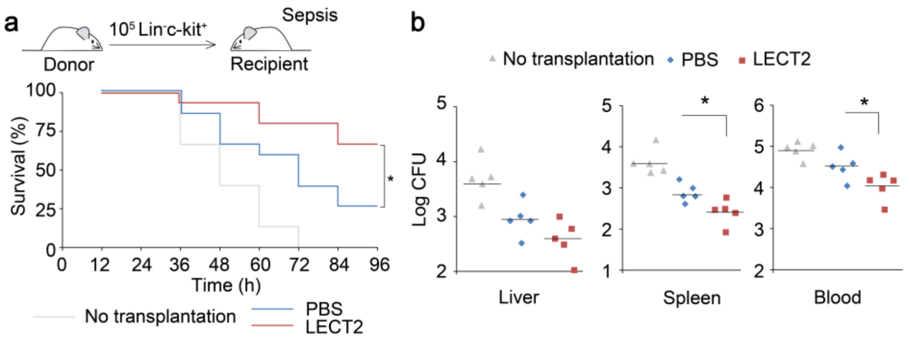

Supplementary Figure 2 LECT2 mobilized HSCs improve the outcome of sepsis. (**a**) The survival rate after PBS- or LECT2-mobilized HSC transplantation in *Pseudomonas aeruginosa*-infected mice. *n* = 15. (**b**) Bacterial clearance was analysed in the liver, spleen, and blood after HSC transplantation. *n* = 5. The data represent means±s.e.m. The data are representative of two independent experiments. $^*P < 0.05$, $^{**}P < 0.01$, $^{***}P < 0.001$ using Kaplan-Meier analysis (**a**) and one-way ANOVA (**b**).

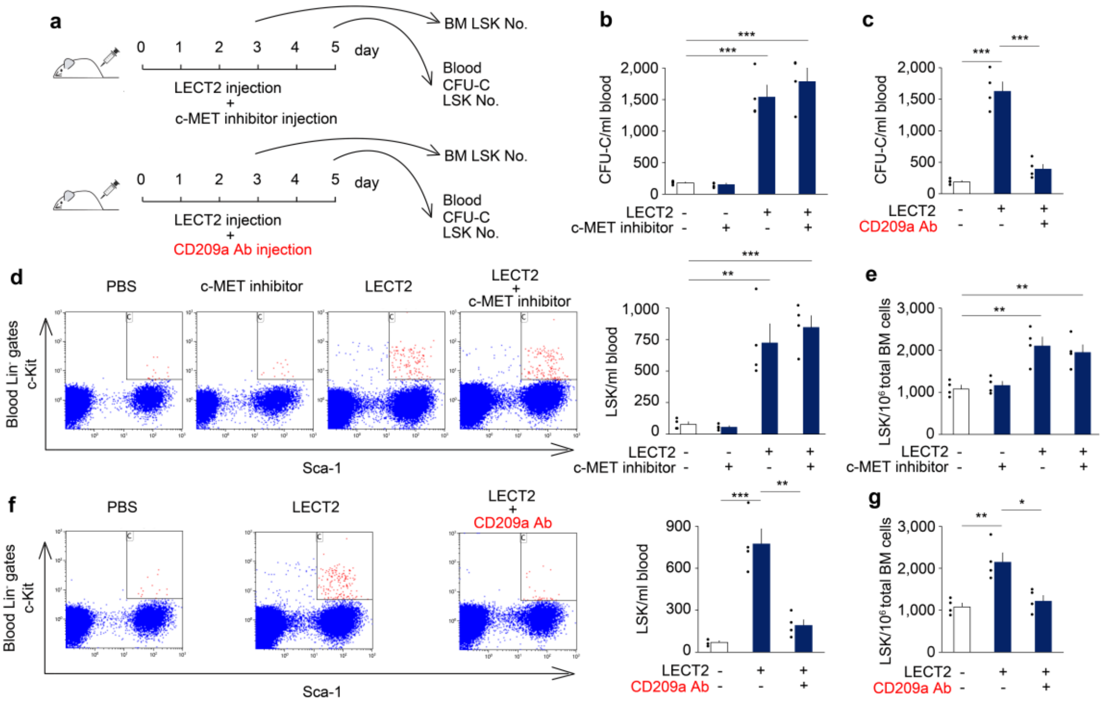

Supplementary Figure 3 CD209a but not c-MET mediates the effects of LECT2 on HSCs. (**a**) Protocol for receptor inhibition. Ab, antibody. (**b,c**) The effects of the c-MET inhibitor and CD209a antibody on CFU-C number in the blood. LECT2 was injected (s.c.) 30 min after the injection (i.p.) of the c-MET inhibitor or CD209a antibody (CD209a Ab). (**d,e**) The effect of the c-MET inhibitor on LSK cell number in the blood and BM after LECT2 treatment. (**f,g**) The effect of the CD209a Ab on LSK cell number in the blood and BM after LECT2 treatment. *n* = 4. The small black dots in histograms are data points. The data represent means±s.e.m. The data are representative of two independent experiments. **P*< 0.05, ***P*< 0.01, ****P*< 0.001 using one-way ANOVA.

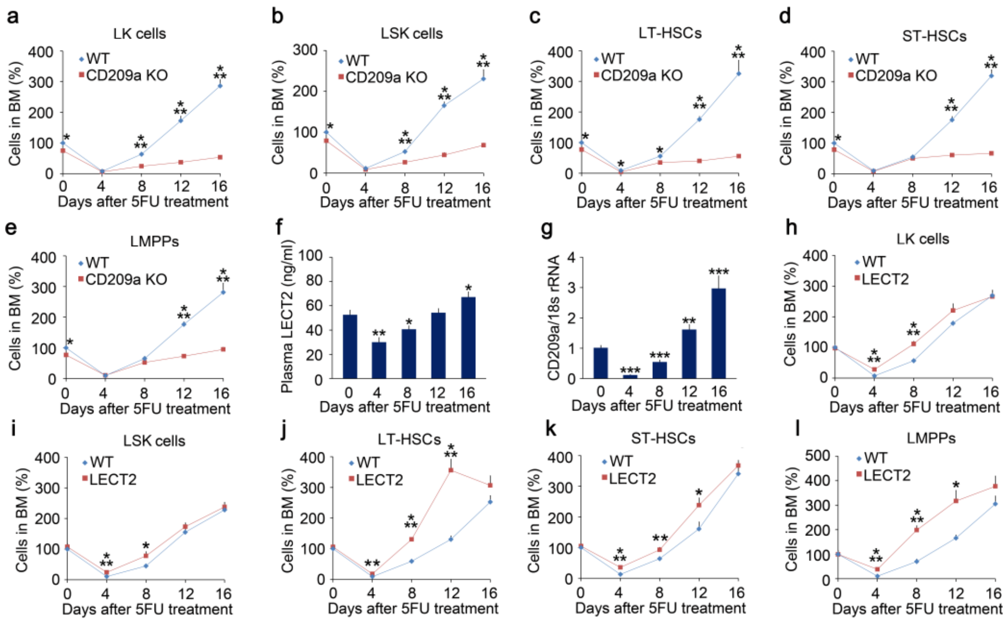

Supplementary Figure 4 LECT2/CD209a signal enhances HSC regeneration after 5FU treatment. (**a-e**) The number of HSCs (LK cells, LSK cells, LT-HSCs, ST-HSCs and LMPPs) in the BM of CD209a KO mice after 5FU treatment. (**f**) Plasma LECT2 levels after 5FU treatment. (**g**) CD209a expression in BM after 5FU treatment. (**h-l**) The number of HSCs in the BM of LECT2 injected mice after 5FU treatment. $n = 5$. The data represent means±s.e.m. The data are representative of two independent experiments. *$P< 0.05$, **$P< 0.01$, ***$P< 0.001$ using one-way ANOVA.

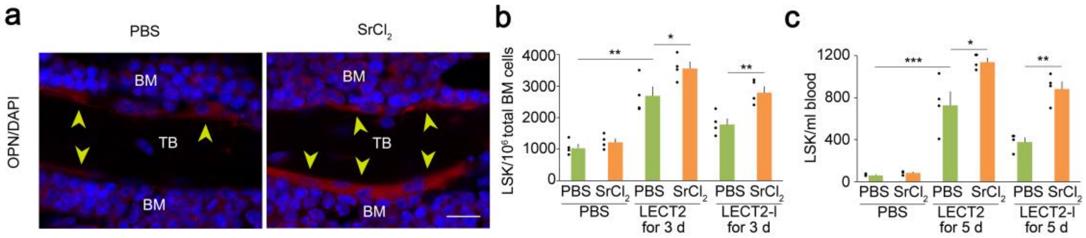

Supplementary Figure 5 The effect of LECT2 on LSK mobilization is enhanced in $SrCl_2$-treated mice. (**a**) Osteopontin (OPN) staining in femur sections from mice treated with $SrCl_2$. Anatomical landmarks are indicated: bone marrow (BM) and trabecular bone (TB). The OPN positive cells are indicated by arrow heads. The scale bar represents 20 μm. The femurs were obtained from control and $SrCl_2$-treated mice. (**b,c**) Quantification of BM and blood LSK cells after $SrCl_2$ treatment. Two doses of LECT2 were used: high-dose LECT2 (300 μg/kg) and low-dose LECT2 (LECT2-l, 100 μg/kg). $n = 4$. The small black dots in histograms are data points. The data represent means±s.e.m. The data are representative of two independent experiments. *$P < 0.05$, **$P < 0.01$, ***$P < 0.001$ using one-way ANOVA.

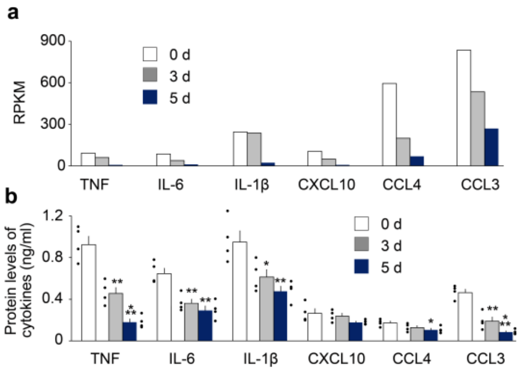

Supplementary Figure 6 LECT2 affects the mRNA and protein levels of cytokines. (**a**) RNA-seq analysis of BM mononuclear cells. The mRNA levels are indicated as Reads Per Kilobase per Million mapped reads (RPKM). (**b**) Changes in cytokine protein levels after LECT2 treatment. The supernatants of the LECT2-treated macrophages were collected for cytokine detection by ELISA. The data are representative of two independent experiments. $n = 4$. The small black dots in histograms are data points. The data represent means±s.e.m. *$P< 0.05$, **$P< 0.01$, ***$P< 0.001$ using one-way ANOVA.

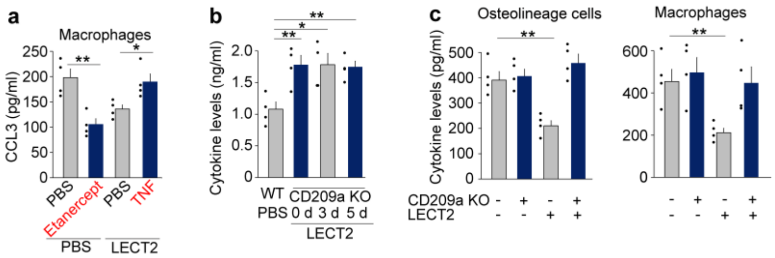

Supplementary Figure 7 TNF production in BM is regulated by LECT2/CD209a signal. (**a**) *In vitro*, CCL3 expression in macrophages after TNF or TNF inhibitor (etanercept) treatment. $n = 4$. (**b**) TNF expression in the BM supernatant after LECT2 treatment of CD209a KO mice. (**c**) LECT2 influences TNF protein levels in osteolineage cells and macrophages *in vitro*. $n = 4$. LECT2 treatment for 48 h. The small black dots in histograms are data points. The data represent means±s.e.m. The data are representative of two independent experiments. *$P< 0.05$, **$P< 0.01$, ***$P< 0.001$ using one-way ANOVA.

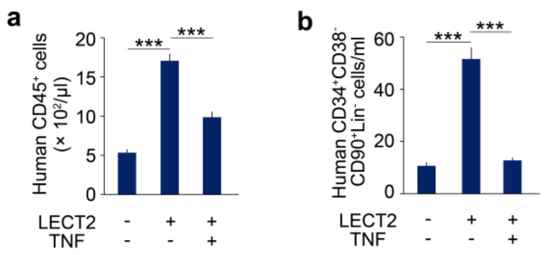

Supplementary Figure 8 TNF mediates the effect of LECT2 on HSC egress in NOD-SCID mice with human HSCs. NOD-SCID mice engrafted with human cells were treated with LECT2 for 5 days with or without the administration of TNF for 2 days. (**a**) The number of $CD45^+$ cells after LECT2 with or without TNF treatment. (**b**) The number of $CD34^+CD38^-CD90^+Lin^-$ cells after LECT2 with or without TNF treatment. The mice were treated with LECT2 for 5 days with or without the administration of TNF for 2 days. n = 5. The data represent means±s.e.m. The data are representative of two independent experiments. ***P< 0.001 using one-way ANOVA.
